# Environmental factors in school classrooms: How they influence visual task demand on children

**DOI:** 10.1371/journal.pone.0210299

**Published:** 2019-01-10

**Authors:** Kalpa Negiloni, Krishna Kumar Ramani, Rachapalle Reddi Sudhir

**Affiliations:** 1 SankaraNethralaya, Unit of Medical Research Foundation, Chennai, India; 2 Shanmugha Arts, Science, Technology & Research Academy (SASTRA) University, Thanjavur, India; 3 Elite School of Optometry, Chennai, India; Yenepoya Medical College, Yenepoya University, INDIA

## Abstract

**Background:**

The key visual factors in a classroom environment include the legibility, angle subtended at the eye, illumination, contrast, and colour of the visual task. The study evaluated the visual environmental factors in the school classrooms.

**Materials and methods:**

The distance Visual Acuity (VA) demand was evaluated based on the size of visual task i.e. the smallest size of chalkboard writing and its viewing distance. The environmental factors which can have an effect on the visibility in classrooms such as illuminance on the chalkboard and at student’s desk, chalkboard contrast, light sources and the student’s perception of their classroom visual environment were measured. To quantify the distance VA demand and to compare with a standard high contrast VA chart measure, a validation of the measurements was performed by chalkboard simulation experiment. The “acuity reserve” to be included to the measured distance VA demand was evaluated.

**Results:**

We included twenty-nine classrooms of eight schools. The median distance VA threshold demand was 0.28 logMAR(0.25,0.45). The median illuminance on front desk position and chalkboard contrast was 130 lux(92,208) and 40(36,50) respectively with 62% classrooms having low illumination (<150lux). The acuity reserve evaluated to be included to the distance VA demand was 0.13logMAR and 0.29 logMAR in classrooms with optimal and low chalkboard illumination respectively which was based on the results of the simulation experiment. The median distance VA demand including the acuity reserve was 0.09 logMAR(-0.03,0.23) [Snellen Equivalent: 20/25(20/19,20/34)].

**Conclusion:**

The study findings highlight the increased visual task demand in school classrooms and the need for appropriate seating arrangements in classrooms based on the visual acuity of children. The study emphasises regular audit of the classroom environment along with the school eye screening.

## Introduction

The critical external factors namely illumination, acoustics, thermal quality, colour and age of school building are typical conditions in determining the quality of a classroom. The poor quality of these characteristics in a classroom can be an environmental stressor increasing strain and subsequently can reduce the academic performance of students [1,2]. Light of different wavelengths can influence various functions such as vision, circadian rhythms, mood, cognition and most importantly the classroom learning and performance [3,4]. Poor lighting reduces visibility and can cause visual discomfortleading to disinterest and lack of concentration. There is a need to understand the classroom visual environment in schools. While the trend of smartboard use is emerging in Indian classrooms, it is far from replacing the conventional chalkboard system (black/ green). Maintenance of good contrast levels on the chalkboard is a prerequisite for better visibility. The Bureau of Indian Standards (BIS) recommends glare index of 16 and lighting level of 150–300 lux on the chalkboard and student desk in a classroom. The evidence of the existing lighting levels in a classroom meeting this stipulation is limited [5,6].

Children spend one-third timein a day at school performing tasks that rely on varying visual demands. Reading and writing from chalkboard is an important visual task in a classroom. The demand imposed on the visual system for better visibility of the visual task is the visual acuity demand and demand for the better performance of the task is the visual task demand. The distance and near visual acuity demand in South Indianschool classrooms and the classroom parameters were evaluated and compared to the recommendations provided by the Indian Standards. An increased distance visual task demand of 20/30 was reported which was based on the writing on the chalkboard and its viewing distance [7]. However, factors such as the illuminance levels, letter legibility, stroke-width and chalkboard contrast can have an effect on the visual acuity measure. This necessitates the estimation and the inclusion of an acuity reserve to advice on distance-visual performance in a school classroom. This study aimed to evaluate the classroom visual environment factors such as the distance visual acuity demandbased on the chalkboard writing and including the acuity reserve, illuminance levels on the chalkboard and at student’s desk, chalkboard contrast and the student’s perception of their classroom visual environment. These visual environmental factors were compared to the visual capability of an individual in the classroom, to estimate the proportion of children under visual stressin their respective classrooms.

## Materials and methods

This is a cross-sectional study conducted in schools located atChennai, South India during August 2014 to February 2015. The classroom visual environmental factorssuch as distance visual acuity demand, chalkboard and desk illumination, light sources, chalkboard contrast and student’s perception of these visual factors were evaluated. We included all the schoolswhich were a part of our regular eye screening programme in schools. This study was approved by the Institutional Review Board and Ethics Committee. We obtained written informed consent from the school authorities and study participants prior to the study.

### Visual acuity demand

**Fig 1:**
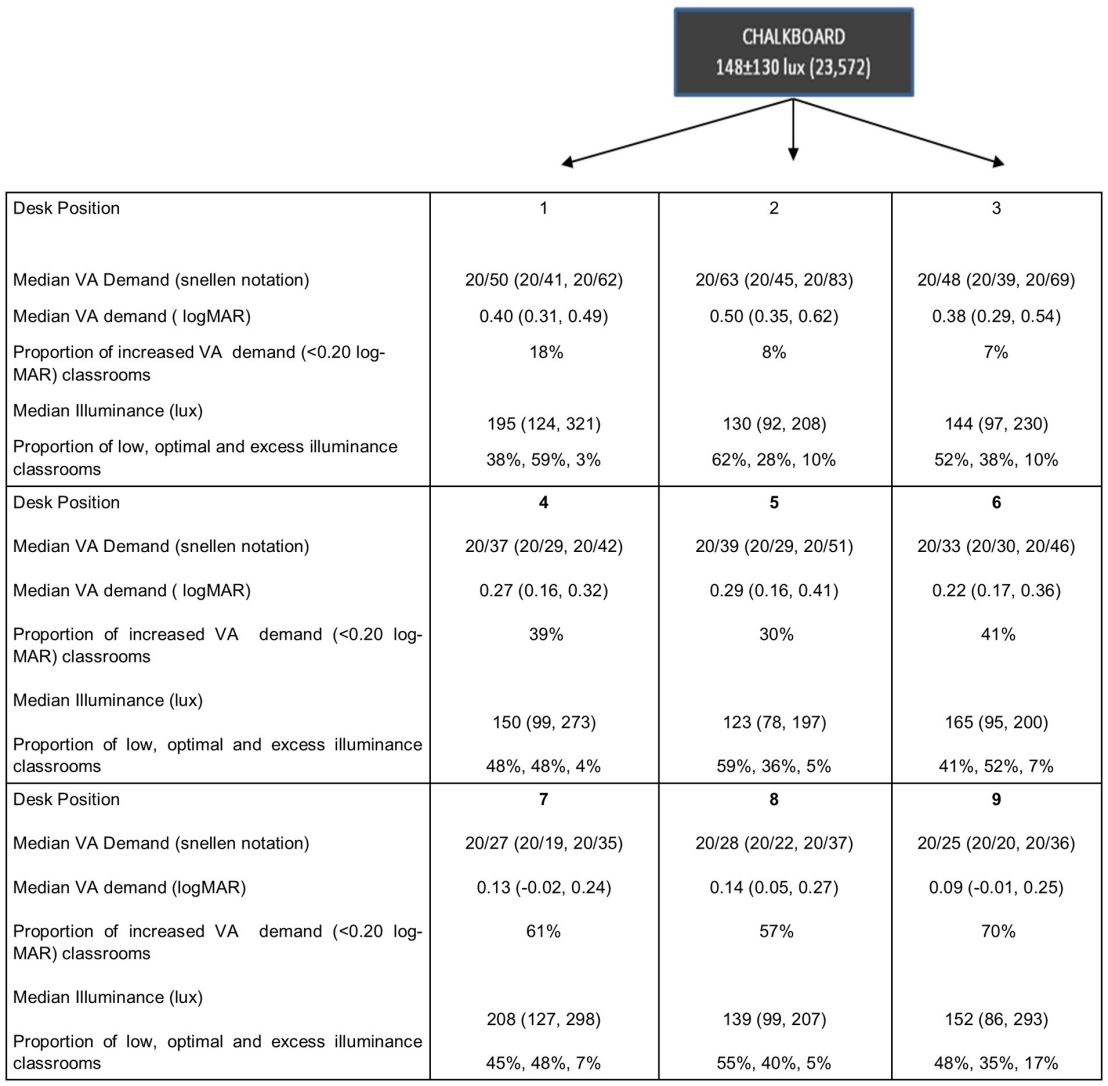
Median visual acuity demand, illuminance levels, proportion of classrooms with increased visual demand and illuminance categories at different desk positions.

One class representing each grade in every school was selected randomly and 29 such classrooms (grade four to 12) of eight schools were included in the study. The distance Visual Acuity (VA) demand was evaluated based on the chalkboard viewing distance and the vertical height of lower case letters of the teacher’s handwriting (in English language only) on the chalkboard applying the same formula and calculation as in previous studies [7,8]. The visual angle subtended at the eye by the visual task was measured based on the viewing distance and visual task size to calculate the snellen and logMAR equivalent distance VA demand. A minimum of 30 letters (centre and side positions) on the chalkboard was measured using a millimetre scale in each of the measured classrooms due to the variability in handwriting (consistency) on the chalkboard. During measurement, care was taken to avoid parallax error. Capital letters and small case letter writing on the chalkboard were excluded and all the measurements were taken by a single observer. The student’s desk was categorised as front, middle and last desk positions (1 to 9) as shown in Fig 1. The viewing distance from the centre of the chalkboard to all these positions was measured thrice and its average was taken. The distance visual acuity threshold demand was evaluated based on the smallest letter size and longest viewing distance in a classroom.

### Chalkboard simulation experiment

To compare the obtained distance VA demand with a high contrast standard visual acuity chart (Early Treatment Diabetic Retinopathy Study, ETDRS logMAR chart) measure, a validation experiment was designed to identify the “acuity reserve” or “conversion factor” under optimal (150–300 lux) and low (<150 lux) [5,6] illumination chalkboard simulation, to advice on the distance visual performance.

The inclusion criteria were subjects with binocular visual acuity better than 20/200 and the presence of low to moderate myopia. Thirty optometry students and optometrists were included with a mean age of 23.7±3.2 years (19, 32). All the subjects had bilateral myopia with mean spherical equivalent power of -1.71±0.68 D (-0.75D, -3.50D) in the eye with low myopia and better unaided visual acuity. The unaided monocular and binocular VA of the subjects was evaluated using an ETDRS logMAR VA chart (Precision Vision, La Salle, Illinois) and recorded. The mean unaided visual acuity in the eye with better visual acuity was 0.72±0.19 logMAR (0.40, 1.00).

#### Experimental set up

A black chalkboard was used for classroom simulation experiment. Lowercase of English alphabets without any ascenders and descenders were selected to fit a 5x5 grid. The height and width of the letter were maintained with a fixed dimension of 3.4cm based on the previous study i.e. the average size of teacher’s writing [7]. A total of three sets with six lower case letters in each were designed. All the subjects were positioned at a farther distance i.e. the distance where the subject cannot recognise any letters written on the chalkboard. The distance between the chalkboard and subjects was initially set at 12 m. The subjects were asked to move slowly and gradually closer towards the chalkboard, maintaining the chalkboard at their eye level, till a point when they could recognise the letters on the chalkboard. The criterion was set as recognising at least four out of six letters. The distance from the chalkboard to the point of recognition of letters was measured and marked as D1. This was repeated thrice with different sets of letters to avoid memorising the letters and the average of the distance was taken. To represent a classroom environment, tube light which was the common artificial light source in schools was used. The uniformity value for illuminance on the chalkboard was calculated as the minimum illuminance value divided by the average illuminance value [9]. The average illuminance level on the chalkboard measured using a Luxmeter was 200 lux (uniformity index: 0.80) for optimal simulation and 90 lux (uniformity index: 0.84) for low illumination simulation.

Based on the viewing distance for a fixed letter height, the chalkboard simulation visual acuity was calculated. Based on the vision level or ETDRS visual acuity of the subject, the actual visual acuity required to view the fixed letter height was evaluated. The difference in visual acuity measure between the actual visual acuity value and chalkboard simulation under optimal and low illumination were calculated. This difference was considered as the “acuity reserve” or “conversion factor” to be included to the measured distance visual acuity demand for the chalkboard under low and optimal chalkboard illumination.

### Illumination and contrast on the chalkboard

The illumination levels on the chalkboard and student’s desk were measured twice during a day (between 8:30–9:30 AM, and between 03:30–04:30 PM) using a calibrated luxmeter (LX 101, Lutron Electronic Enterprise Co., Ltd, Taiwan). All the measurements were taken on a bright sunny day (30° to 34°C). The uniformity value for illuminance on the chalkboard and student’s desk was calculated as the minimum illuminance value divided by the average illuminance value [9]. The total number and positioning of the doors, windows and artificial light source (including working condition) was documented for each classroom. A calibrated photometer (PR-655 Spectrascan Spectroradiometer, Photo Research Inc., Chatsworth, CA) was used to measure the luminance levels (quantified in Cd/m^2^) of the letter on the chalkboard and its background. The measurements were repeated twice, during the morning (first lecture hour, AM) and afternoon (cleaning the chalkboard and minimum of three-time chalkboard use, PM). The contrast levels were represented as Weber contrast (Luminance (L) contrast = L_background_- L_target_/ L_background_). The student’s desk was categorised as front, middle and last desk positions (1 to 9) as shown in Fig 1. The illuminance levels and the visual acuity demand at these desk positions were evaluated.

### Student’s perception of classroom environment

The survey of student’s perception of chalkboard visibility, lighting levels on their desk, the presence of glare and contrast levels on the chalkboard was evaluated by administering a four-item structured (closed-ended) questionnaire (S1 Text). The survey questions included visibility on the chalkboard, lighting levels, glare and, chalkboard contrast. Students were queried regarding the presence or absence of glare on board and the position of glare (centre or side position) on the chalkboard if present. The illumination levels (lighting) on the desk perceived by students was categorised as poor, normal or excess lighting. The contrast of letters on the chalkboard were categorised as good or poor contrast. Questions were asked in English and alsoexplained in the local vernacular language (Tamil) if questions were not clear. Details regarding the seating positions of these students in their respective classroom were documented.

### Evaluation of visual stress in classrooms

The distance visual acuity demand when children are seated at different desk positions in their respective classrooms was evaluated. The acuity reserve was included to the measured VA demand based on low or optimal illuminance measures on the chalkboard in the respective classrooms.

The average class size of 29 classrooms included in the study was 36±10 children (20, 58). A total of 1038 children (51% girls) were present in the measured 29 classrooms. Based on the report of vision screening performed in these schools, the distance visual acuity was measured using a Pocket vision screener (PVS) with a cut off visual acuity level of 0.20 logMAR or 20/30. The vision screening report noted only one child of 1038 children having best corrected visual acuity of 20/200 in both the eyes. All the other children had best corrected visual acuity of better than 20/30. Based on the vision levels of children, the proportion of children under visual stress in their respective classrooms was evaluated. “Visual stress” was defined as “visual requirement in excess of what the child has”. The risk of visual stress in classrooms was measured by calculating the proportion of classrooms with visual acuity demand worse than 20/30. We analysed the risk of visual stress at different desk position in classroom with the 20/30 cut off visual acuity.

### Statistical analysis

All the data was entered in Excel (Microsoft office 2013) and the statistical analysis was performed using IBM Statistical Package for Social Sciences (SPSS) version 20. Descriptive analysis of the classroom environment variables was performed and the values were presented as median (interquartile range 25^th^ and 75^th^ percentile). Non-parametric tests were performed and p-value less than 0.05 was considered as statistically significant.

## Results

### Visual acuity demand and chalkboard simulation experiment

The median distance visual acuity threshold demand based on the smallest size of the chalkboard writing and longest viewing distance was 0.28 logMAR (0.25, 0.45) in twenty nine classrooms of eight schools (S1 Table). Based on the chalkboard simulation experiment, the median visual acuity under optimal and low illumination chalkboard simulation was approximately one and a half logMAR lines (Median: 0.13 logMAR) and three logMAR lines (median: 0.29 logMAR) less than the actual ETDRS acuity and the difference was statistically significant (optimal; p = 0.008 and low; p<0.001). After including the acuity reserve, the median visual acuity threshold demand was 0.09 logMAR (-0.03, 0.23). The median VA demand at front desk position was 0.50 logMAR (0.35, 0.62).

### Illumination and contrast on the chalkboard

The illuminance level and the uniformity index on the chalkboard are presented in Table 1. The median illuminance level on the chalkboard and student’s desk positions (1 to 9) categorised based on low (<150 lux), optimal (150–300 lux) and excess (>300 lux) levels are presented in Table 2 and Fig 1. In each of the observed classrooms, on an average twofluorescent lamps (range, 1–6 tube lights), twowindows (range, 1–4) and onedoor (range, 1–2) were theavailable light sources. Five classrooms had no artificial light source and were dependent on three to four windows and one door for lighting. Seven classrooms with 3–4 tube lights and 1–2 lights were found in non-working/ unrepaired condition. The mean illuminance level on the front desk position in the five classrooms without artificial light source was 204±150 lux (range, 47–435). The mean illuminance level of students sitting beside window was 313±276 lux (range, 45–1252), directly below tube light was 243±177 (57–816), and in other positions it was 152±165 lux (22–1210).

**Table 1:** The median illuminance and luminance levels on the chalkboard and student’s desk during morning and afternoon measurements.

Variables	Morning (AM)		Afternoon (PM)		
	Median (IQR)	Range (minimum, maximum)	Median (IQR)	Range	AM and PM difference (p value)
Chalkboard centre Illuminance (lux)	104 (55, 175)	23, 572	133 (70, 203)	26, 490	<0.001
Uniformity index of chalkboard	0.74 (0.56, 0.82)	0.42, 0.91	0.71 (0.55, 0.79)	0.19, 0.87	0.234
Uniformity index of student’s desk	0.56 (0.42, 0.68)	0.27, 0.88	0.54 (0.38, 0.71)	0.16, 0.82	0.449
Luminance of chalkboard (L_background_) Cd/m^2^	1.28 (0.95, 1.67)	0.39, 28	1.43 (1.05, 1.92)	0.61, 28	0.008
Contrast level (Weber)	40 (36, 50)	20, 70	36 (30, 49)	21, 59	0.088

IQR—Interquartile range. Descriptive information on the range is provided to present the maximum and the minimum limit of the variables

**Table 2:** The median illuminance levels (lux) at desk positions 1–9 during two different measurements (Morning and afternoon).

Category of desk position	Desk position	Morning (AM)		Afternoon (PM)		AM and PM (p value)
		Median illuminance (IQR)	Range (Min, Max)	Median illuminance (IQR)	Range (Min, Max)	
Front row	1	195 (124, 321)	41, 586	180 (107, 354)	52, 1472	0.124
	2	130 (92, 208)	45, 699	136 (90, 252)	50, 1507	0.164
	3	144 (97, 230)	47, 1235	170 (110, 259)	48, 721	0.028
Middle row	4	150 (99, 273)	31, 816	170 (99, 272)	20, 1022	0.030
	5	123 (78, 197)	43, 563	128 (87, 213)	45, 1040	0.159
	6	165 (95, 200)	40, 1252	173 (82, 220)	46, 730	0.094
Last row	7	208 (127, 298)	22, 908	171 (113, 268)	25, 1120	0.309
	8	139 (99, 207)	35, 518	130 (92, 204)	39, 980	0.935
	9	152 (86, 293)	37, 1252	150 (80, 279)	48, 850	0.729

IQR- Interquartile range. Descriptive information on the range is provided to present the maximum and the minimum limit of the variables

The contrast of letters on the chalkboard during first and last lecture hour (cleaning the chalkboard and minimum use of the chalkboard, 3–5 times) was assessed in 10 classrooms of four schools. The mean luminance levels on the surface of the board (L_background_) measured during the start of the day (first lecture hour) was 1.30 ± 0.59 Cd/m^2^ (0.39, 3.52) and during the last lecture period was 1.46 ± 0.56 Cd/m^2^(0.61, 3.98). The mean Weber contrast during the first lecture was 43 ± 13 (20, 71) and 39 ±11 (21, 59) during last lecture hour and was statistically insignificant (p = 0.440). There was statistically no significant difference in the Weber contrast between 10 measured classrooms between the first (p = 0.994) and the last measurement (p = 0.928). In a newly painted blackboard, the mean Weber contrast was 71 ± 4 (66, 77).

### Student’s perception of classroom environment

**Fig 2:**
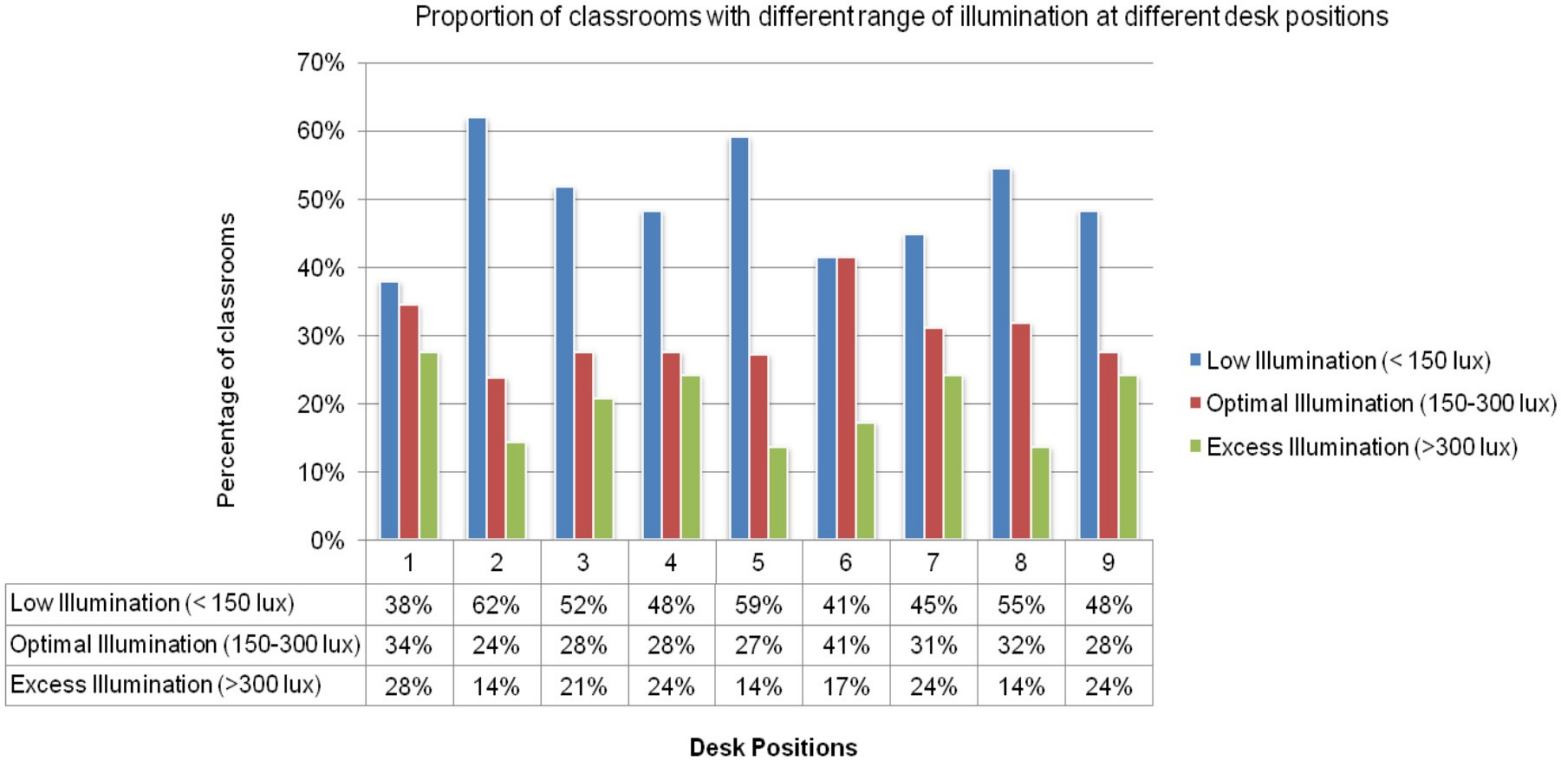
Categorization of the illumination levels at different desk positions and proportion of classrooms with different range of illumination.

A total of 343 children (grades four to ten from 12 classrooms) responded to the survey questions. Twenty-eight (8%) children reported that the letters on the chalkboard were not visible in their respective classrooms from their seating (desk) position. Among them, four children were noted to have uncorrected refractive error. Regarding illumination level, 13% (45 out of 343) children perceived their desk illumination to be poor, 83% (285 out of 343) as normal lighting and 4% (13 out of 343) children reported excess lighting. Based on the categorisation of actual illuminance on student’s desk, 54% (185 out of 343) desk positions had illuminance level less than 150 lux, 42% (143 out of 343) had illuminance between 150–500 lux and 4% (15 out of 343) had illuminance more than 500 lux. The actual illuminance level was compared to student’s perception of lighting levels on their desk position as shown in Fig 2. The presence of glare on the chalkboard was reported by 26% (90 out of 343) children, with 72% (n = 65) reporting glare on the sides of the chalkboard. A total of 208 children responded to symptom survey related to contrast. Among them, 28% of students perceived chalkboard to have poor contrast levels in their classrooms (Actual mean contrast: 41±11) and 72% perceived the boards in their respective classrooms to have good contrast levels (Actual mean contrast: 46±11).

### Visual stress in classrooms

**Fig 3:**
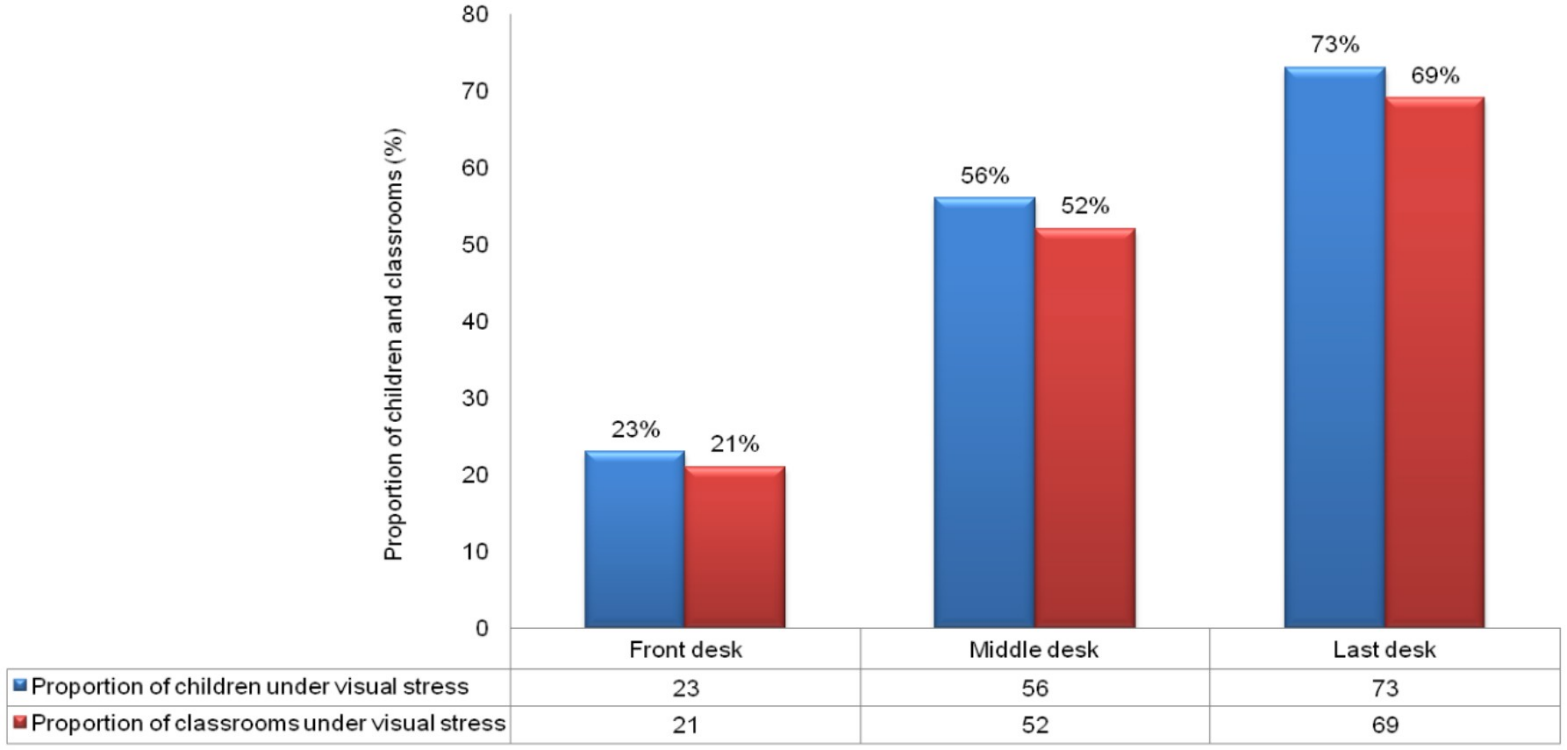
Proportion of children and classroom under visual stress categorised based on front, middle and last desk position. Proportion of children under visual stress in different desk positions is based on comparing the visual acuity level of children and their respective classroom visual demand.

The visual acuity demand and proportion of classrooms with increased demand(> 20/30), when children are seated at different desk positions are presented in Fig 1. Optimal illuminance level (150–300 lux) on the chalkboard was noted in 31% classrooms (n = 9) and the remaining 69% classrooms (n = 20) had low illumination level (<150 lux). Overall, nine classrooms had VA demand greater than 20/30 causing visual stress in children. The proportion of children and classrooms under the risk of visual stress when seated at different desk positions (front, middle and last) is presented in Fig 3. A logistic regression was performed to ascertain the effect of student desk position (front, middle and last row) on having visual stress. We found that the children seated in middle row had an Odds of 4.34 (95% CI 3.592, 5.254, p<0.05) times risk of visual stress when compared to children seated in front row. Similarly, when seated in last row they had an Odds of 9.12 (95% CI 7.467, 11.129, p<0.05).

Proportion of classrooms under visual stressin different desk positions is based on comparing the classroom visual acuity demand and cut off acuity demand level of 20/30.

## Discussion

This study highlights the existing levels of the school classroom visual environmental factors. The study recommends an acuity reserve of one and a half lines (0.13 logMAR) and three logMAR lines (0.29 logMAR) to be included to the distance visual acuity demand evaluated for chalkboard writing under optimal (150–300 lux) and low (<150 lux) illumination respectively to compare with a standard visual acuity chart measure.

An increased average distance visual acuity demand of 20/25 was noted in classrooms which were based on the smallest chalkboard writing and longest viewing distance in the classroom. The average visual acuity demand was 20/63 for children seated in the front desk of the classrooms. The findings highlight the presence of increased visual task demand in viewing the chalkboard in school with optimal and low chalkboard illuminance levels.

Good lighting is an essential component in designing a school building. The Government of India recommends standard lighting design for Educational Buildings, especially schools, as the premises are used for awide range of activities embracing a complex array of different visual tasks. The study results highlight that illuminanceof less than the standard recommendation of 150 lux was noted on student’s desk in more than 38% classrooms. The illuminance levels werelower in the middle desk positions in the classroom as compared to other desk positions. This could be due to the positioning of light sources (artificial and natural light). Children with poor vision, slow learners, or other difficulties are usually placed in the front desk position in a classroom. The illuminance front desk position on 62% of the classrooms in the study had lower illuminance levels, which was consistent with previous research. A study conducted in schools of Dharward, India reports that the classroom interior standards, including the illumination, were found to be lower than the BIS recommendation [10]. The levels of uniformity in illuminance level of chalkboard and desk in most of the classrooms were below the recommended value of 0.8 [9,11]. Variation in daily weather conditions can affect the measured illuminance levels. The current study limited itself only to lighting measurement performed on a single day. However, the data collection was performed on a bright sunny day and measurements were taken twice in a day. The classroom interiors need to be redesigned to provide uniform illuminance levels on chalkboard and students desk. The ancient to 20^th^century school classrooms have always been designed with rings or rows of seats to focus the attention of many towards one. The difference has been open classroom lit with natural sunlight as opposed to closed rooms with an additional artificial light source. Good lighting has a positive effect ona student’s learning and performance in a classroom. Classrooms are now dependent on artificial light sources in addition to the natural light sources. With technological improvement, light source designed for school vision has been found to increase reading speed by 35%, decrease in frequency of errors by 45% and the decrease in hyperactive behaviour by 76% and saving energy up to 57% [12]. One of the major public health problems globally studied is the myopia which has genetic and environmental risk factor. The prevalence of myopia in children has significantly increased in Asian countries, including in India [13]. The important environmental risk factors being studied are increased near work, less outdoor, visual environment and ergonomics, and urbanisation [13]. Hua et al found that higher ambient light level is a protective factor for non-myopic students on myopia onset and retarded axial growth in myopic and non-myopic students [9]. Norton and Siegwart based on their detailed review of literature proposed a model supporting optimal illuminance levels as a protective factor for normal refractive development and lower light levels for causing myopia progression possibly due to the elevation of retinal dopamine activity [14]. Zhou et al designed a bright classroom with an aim of resembling the light conditions to outdoors and to prevent myopia progression [15]. Children and teachers rated better reading comfort at different intensities in bright classrooms compared to the traditional classrooms.

In school classrooms, the contrast of letters on conventional board system (black/green chalkboard) is an important factor for better visibility. The contrast reduction due to ocular disorders and reduced contrast of the task material, both have an effect on visibility and learning. Decreased contrast can reduce visual span, reading speed and saccade size in reading. BIS recommends the chalkboard to be painted matt black or green with reflection factor maintained between 15–30% to ensure easy readability from any point in a classroom [5,6]. The current study reported no difference in the contrast levels with cleaning and using the chalkboard several times during a day. The contrast measure may be affected by various factors such as the illuminance on the surface of the board, reflectance property based on the thickness of the chalk material and the period of board use. The current study used a calibrated and reliable photometer for the best possible contrast measure. In one of the classrooms, a newly painted blackboard had the contrast range of 66 to 77, highlighting the influence and need to maintain recommended luminance on the board for better visibility.

An interesting finding in the current study was that students sitting in desk position with low illuminance levels (less than 150 lux) perceived and reported lighting levels were normal (60%)or excess (9%) at their desk. This could be due to the adaptation of the visual system under different illumination through a coordinated action of the mechanical, photochemical and neural process in the visual system. This cannot be generalised considering the response bias and understanding of normal illuminance level by children. Presence of glare on the side portions of the chalkboard reported may be due to the reflections from the doors and windows which are generally positioned in the corners of a classroom. Further study on the effect of a range of light levels on the visual system would aid providing a better visual classroom environment for children without visual stress and discomfort.

An increased visual acuity demand in viewing the chalkboard is reported by including the acuity reserve based on the chalkboard illumination. The study also highlights that the visual acuity demand did not match the vision levels of children, causing visual stress. We report 23% of children in front desk and 73% seated in last desk position did not meet the visual demand in their respective classroom. Even children with normal visual acuity or better than 20/30 acuity were at risk of visual stress in their classrooms when seated at last desk position. Children with visual impairment or low vision when seated in front desk were at risk of visual stress with an average visual demand of 20/48 acuity. This suggests the need for visual environmental modifications in school classrooms. Children with mild to moderate visual impairment can be advised on the size of chalkboard writing and seating position in a classroom to continue mainstream schooling. A recent study on children with “mild visual impairment” and range of “20/400 to 20/200” visual acuity, suggest a maximum of 4.3m and 85cm to 1.7m as the viewing distance from the chalkboard to view the chalkboard writing with a minimum size of 3cms and 4cms respectively [16].

## Conclusion

An increased visual acuity demand was noted in school classrooms with varying lighting and contrast demand. Majority of classrooms had measures below the standard recommendations. Children with normal visual acuity in few of the measured classrooms did not meet the visual acuity demand in their respective classrooms. Further assessment of the effect of classroom visual factors in children with visual anomalies may provide additional important information. The inclusion of acuity reserve based on the type of classroom illumination can aid a clinician to understand the chalkboard visual acuity demand and recommend classroom modifications for varying levels of visual acuity of children. The children with visual impairment can be advised on the classroom environmental modifications and can continue in mainstream schools. This study also highlights the need for regular audit of environmental variables of the school classrooms along with school eye screening.

## Supporting information

S1 Text Survey questions. (DOCX)

S1 Table Visual environmental factors in school classroom. (XLSX)
