# Supplementary material for: Environmental factors in school classrooms: How they influence visual task demand on children
Source: PLoS One. 2019 Jan 10;14(1):e0210299. doi: 10.1371/journal.pone.0210299 (PMC6328081; doi:10.1371/journal.pone.0210299)
Supplement: S1 Text — (DOCX) [file pone.0210299.s001.docx]

**Symptom survey questions**

Name: Date:

Class: Section:

Seating position in classroom: Chalkboard positions:


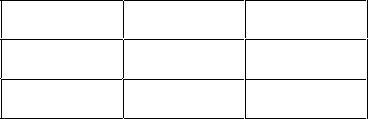

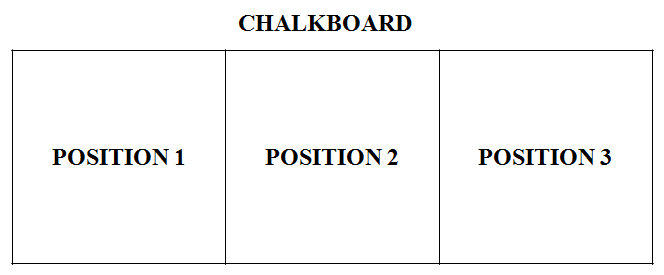


| S.No | Question | Please tick ( ) the right option | | |
| --- | --- | --- | --- | --- |
| 1 | Can you see the teacher’s handwriting on the chalkboard? | Yes |  | No |
|  | If not visible, which chalkboard position is not clear? | Position 1 | Position 2 | Position 3 |
| 2 | Do you go closer to the chalkboard to copy notes | Yes |  | No |
| 3 | Do you see glare on the chalkboard | Yes |  | No |
|  | If yes, which position on the chalkboard has glare | Position 1 | Position 2 | Position 3 |
| 4 | How is the lighting level on your desk | Poor | Normal | Excess |
| 5 | How is the contrast of writing on the chalkboard | Good |  | Poor |
